# Risk Stratification for Diffuse Large B-Cell Lymphoma by Integrating Interim Evaluation and International Prognostic Index: A Multicenter Retrospective Study

**DOI:** 10.3389/fonc.2021.754964

**Published:** 2021-12-16

**Authors:** Xue Shi, Xiaoqian Liu, Xiaomei Li, Yahan Li, Dongyue Lu, Xue Sun, Ying Li, Shunfeng Hu, Yuanfeng Zhang, Xiangxiang Zhou, Xin Wang, Haiping Chen, Xiaosheng Fang

**Affiliations:** ^1^ Department of Hematology, The Affiliated Hospital of Qingdao University, Qingdao, China; ^2^ Department of Hematology, The Affiliated Yantai Yuhuangding Hospital of Qingdao University, Yantai, China; ^3^ Dongying People’s Hospital, Medical Records Department, Dongying, China; ^4^ Department of Hematology, Shandong Provincial Hospital Affiliated to Shandong First Medical University, Jinan, China; ^5^ Department of Hematology, Shandong Provincial Hospital, Cheeloo College of Medicine, Shandong University, Jinan, China; ^6^ School of Medicine, Shandong University, Jinan, China; ^7^ Department of Infectious Diseases, Shandong Provincial Hospital Affiliated to Shandong First Medical University, Jinan, China

**Keywords:** diffuse large B-cell lymphoma, International Prognostic Index, risk stratification, prognosis, interim evaluation

## Abstract

The baseline International Prognostic Index (IPI) is not sufficient for the initial risk stratification of patients with diffuse large B-cell lymphoma (DLBCL) treated with R‐CHOP (rituximab plus cyclophosphamide, doxorubicin, vincristine, and prednisone). The aims of this study were to evaluate the prognostic relevance of early risk stratification in DLBCL and develop a new stratification system that combines an interim evaluation and IPI. This multicenter retrospective study enrolled 314 newly diagnosed DLBCL patients with baseline and interim evaluations. All patients were treated with R-CHOP or R-CHOP-like regimens as the first-line therapy. Survival differences were evaluated for different risk stratification systems including the IPI, interim evaluation, and the combined system. When stratified by IPI, the high-intermediate and high-risk groups presented overlapping survival curves with no significant differences, and the high-risk group still had >50% of 3-year overall survival (OS). The interim evaluation can also stratify patients into three groups, as 3-year OS and progression-free survival (PFS) rates in patients with stable disease (SD) and progressive disease (PD) were not significantly different. The SD and PD patients had significantly lower 3-year OS and PFS rates than complete remission and partial response patients, but the percentage of these patients was only ~10%. The IPI and interim evaluation combined risk stratification system separated the patients into low-, intermediate-, high-, and very high-risk groups. The 3-year OS rates were 96.4%, 86.7%, 46.4%, and 40%, while the 3-year PFS rates were 87.1%, 71.5%, 42.5%, and 7.2%. The OS comparison between the high-risk group and very high-risk group was marginally significant, and OS and PFS comparisons between any other two groups were significantly different. This combined risk stratification system could be a useful tool for the prognostic prediction of DLBCL patients.

## Introduction

Diffuse large B-cell lymphoma (DLBCL) is the most common type of non-Hodgkin’s lymphoma (NHL) and accounts for about 30%–40% of all NHL cases (1). This heterogeneous disease can be subdivided into several types according to different manifestations and molecular characteristics using the WHO classification (2). Even though the survival rate has been improved by immunochemotherapy advances in the last two decades (3), 30%–40% of DLBCL patients experience relapse or refractory disease. It is therefore important to promptly identify DLBCL patients who are unlikely to be cured with first-line immunochemotherapy and develop personalized therapy strategies.

Several prognostic score systems have been established and applied to predict the survival of patients with DLBCL. The International Prognostic Index (IPI) is a well-established prognostic index system. It identifies four independent risk groups of patients with a combination of five clinical variables including age, serum lactate dehydrogenase (LDH) level, tumor stage, Eastern Cooperative Oncology Group (ECOG) Performance Status (PS), and extranodal sites of disease (4). The IPI has been widely used in clinical applications and is the standard practical prognostic tool for DLBCL patients. However, it was established before the immunochemotherapy era, which has dramatically increased the survival rate. According to various studies, the IPI system failed to identify a high-risk group after immunochemotherapy (5–8).

Several other prognosis evaluation systems have been developed for more precise evaluation of DLBCL patients. The National Comprehensive Cancer Network (NCCN)-IPI system is generally based on the IPI and further considers the influence of different ages and LDH levels. Compared with the IPI, the NCCN-IPI has enhanced discrimination power for both low- and high-risk patients in the immunochemotherapy era (9). However, two studies found that NCCN‐IPI high‐risk patients still had quite a high progression-free survival (PFS) rate of 40%–60%, which indicated a need for better classification of high-risk patients (10, 11). Since the NCCN-IPI was mainly based on western populations, its stratification values are still challenged by other studies, especially those performed in eastern populations.

The IPI and NCCN-IPI are both based on baseline disease characteristics. DLBCL can also be divided into subgroups based on different cells of origin and molecular characteristics that influence chemotherapy sensitivity and patient survival (12). DLBCL patients are traditionally evaluated after 2–4 regimens, and residual disease after treatment also indicates prognosis. Several studies found that interim evaluation results from PET-CT or CT scans are predictors of survival (13–15), but given the considerable portion of false-positive PET-CT scans, interim evaluation is not strictly associated with survival.

Here, we developed a prognosis evaluation system by integrating interim evaluations based on CT/PET-CT examinations and the IPI system to more precisely predict the survival of patients with DLBCL.

## Materials and Methods

### Study Cohort

We retrospectively investigated the clinical data of 314 adult patients diagnosed with DLBCL not otherwise specified (NOS) at the Shandong Provincial Hospital affiliated to the First Medical University of Shandong, the Affiliated Hospital of Qingdao University, the Affiliated Yantai Yuhuangding Hospital of Qingdao University, and Dongying People’s Hospital from December 2008 to December 2019. All enrolled patients had adequate clinical information available for analysis. Baseline clinical information required for the study was age, Ann Arbor stage, serum LDH level, ECOG-PS score, and extranodal involvements. All patients received 6–8 cycles of R-CHOP (rituximab plus cyclophosphamide, doxorubicin, vincristine, and prednisone) and R-CHOP-like regimens as the first-line treatment. After the initial 2–4 immunochemotherapy cycles, all patients underwent CT or PET-CT imaging examination for interim evaluation.

The study was conducted in accordance with the Declaration of Helsinki and approved by the Medical Ethical Committee of the Shandong Provincial Hospital affiliated to the First Medical University of Shandong, the Affiliated Hospital of Qingdao University, the Affiliated Yantai Yuhuangding Hospital of Qingdao University, and Dongying People’s Hospital.

### CT and PET-CT Procedures

All patients underwent CT or PET-CT scanning of the neck, chest, abdomen, and pelvis prior to the first immunochemotherapy cycle (baseline evaluation) and after 2–4 cycles of treatments (interim evaluation). PET-CT scans were generally scheduled the third week after the prior 2–4 cycles of immunochemotherapy, but they were postponed if granulocyte colony-stimulating factor was administered within 48 h of the scheduled scan. Patients’ blood glucose levels were required to be <11 mmol/L, and patients fasted for at least 6 h prior to intravenous injection of ^18^F-fluorodeoxyglucose (^18^F-FDG).

### International Prognostic Index and Interim Assessment

The IPI score and interim assessment were retrospectively assessed in patients who had complete data for all variables. The interim responses were divided into four groups as complete remission (CR), partial response (PR), stable disease (SD), and progressive disease (PD) according to the Lugano Response criteria (16).

### Statistical Analysis

The primary purpose of this study was to evaluate the association between prognosis and the new risk stratification system that combined interim evaluation data and baseline IPI. PFS was defined as the time from the date of diagnosis to the date of first documented progression of disease or death, and overall survival (OS) was defined as the time from the date of diagnosis to the date of death from any causes or the last follow-up. The 3-year PFS and OS rates were calculated using the Kaplan–Meier method, and log-rank tests were used to identify differences in the variables. All data analyses were carried out in SPSS version 20.0 (IBM Corp., Armonk, NY, USA). Differences were considered significant at p < 0.05.

## Results

### Patient Characteristics

A total of 314 newly diagnosed patients were enrolled in the study. The median age was 52 (range 11–84), 129 (41%) patients were older than 60 years, and there was a slight predominance of males (164/314, 52%). Most patients (193/314, 61.5%) had normal LDH levels. Overall, 75.2% (236/314) of patients were diagnosed with advanced stage (stages III–IV), and 29.9% (94/314) of patients had B symptoms; 37.9% (106/280) were germinal center B-cell (GCB) type, and 62.1% (174/280) were non-GCB type according to Hans Criteria. All enrolled patients were effectively followed up for a mean period of 30.3 months (range 1–80 months) (**Table 1**).

**Table 1:** Clinical characteristics of patients at diagnosis (n = 314).

Variables	n (%)
Age >60 years	129 (41)
Males	164 (52)
Normal LDH level	193 (61.5)
Ann-Arbor stage (III–IV)	236 (75.2)
B symptom	94 (29.9)

LDH, lactate dehydrogenase.

### Outcomes of the Entire Cohort According to International Prognostic Index and Interim Evaluation

**Figure 1:**
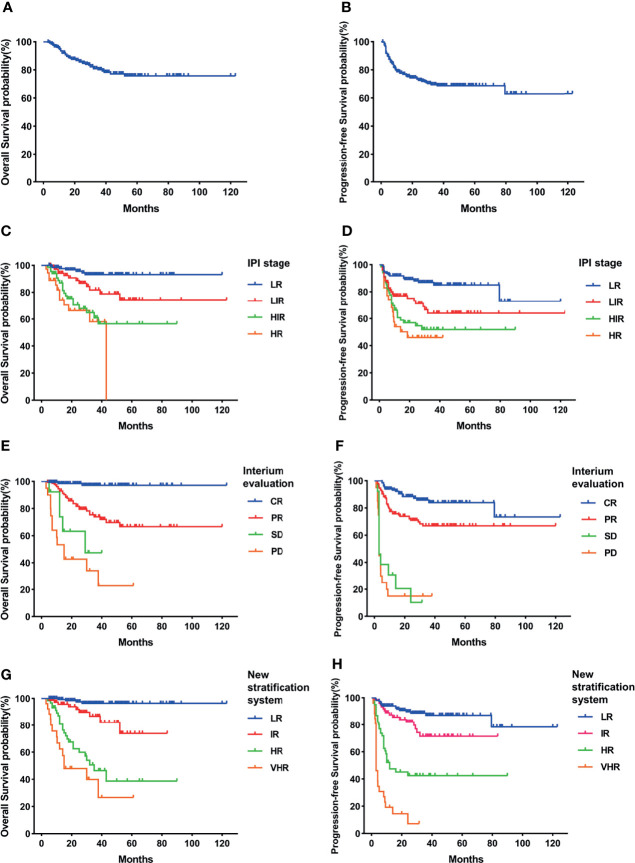
Survival rates of the total cohort. **(A)** Overall survival (OS). **(B)** Progressive-free survival (PFS). **(C)** OS according to International Prognostic Index (IPI) and interim evaluation. **(D)** PFS according to IPI and interim evaluation. **(E)** OS when stratified with interim evaluation. **(F)** PFS when stratified with interim evaluation. **(G)** OS according to the new risk stratification system by combination of interim evaluation and IPI. **(H)** PFS according to the new risk stratification system by combination of interim evaluation and IPI.

At a median follow-up of 30.3 months, 263 (75.7%) patients had survived. The 3-year OS and PFS rates for the total cohort were 80.1% and 68.7%, respectively (**Figures 1A, B**).

When stratified with the IPI, 135 (43%) patients were low risk, 76 (24.2%) were low-intermediate risk, 68 (21.7%) were high-intermediate risk, and 35 (11.1%) were high risk. The estimated 3-year OS was 93.1% for the low-risk (LR) group, 86.7% for the low-intermediate-risk (LIR) group, 75.4% for the high-intermediate-risk (HIR) group, and 57.3% for the high-risk (HR) group (**Figure 1C**). The 3-year PFS rates of each group were 85.1%, 75.1%, 51.9%, and 46.0% (**Figure 1D**). The HIR and HR groups had overlapped survival curves with no significant difference (p = 0.46). Based on this, the IPI could stratify patients into three independent risk groups according to OS and PFS. Moreover, even the HR group still had >50% long-term survival, suggesting that this approach was limited in identifying the real HR group in our cohort.

When stratified using interim evaluation results, 43% (135/314) of patients achieved CR, 46.5% (146/314) achieved PR, 4.1% (13/314) had SD, and 6.4% (20/314) had PD. Patient survival also varied according to interim evaluation (**Figures 1E, F**). CR patients had the highest percentages of 3-year OS (97.1%) and PFS (84%), while the survival curves of SD and PD patients overlapped and showed the lowest survival incidence (3-year OS, 47.5% and 34.1%, respectively; 3-year PFS, 10.3% and 15% respectively). PR patient survival rates were between those of the CR and SD/PD patients (3-year OS of 73.9% and 3-year PFS of 67%), which were significantly higher than those of the SD or PD group (p < 0.05) and lower than those of the CR group (p < 0.05). Like the IPI, interim evaluation results could also separate the cohort into three independent risk groups. However, with this classification, we found that the percentage of SD and PD patients was only 10.5%, again suggesting limitations in identifying HR patients.

### Outcome of the Entire Cohort According to the New Risk Stratification System by Combining Interim Evaluation and International Prognostic Index

Even though the above two approaches could stratify patients into different risk groups, neither is sufficient for prognosis. For more useful risk stratification, we established a new system by combining the interim evaluation results and IPI (**Table 2**).

**Table 2:** The new stratification system combining IPI and interim evaluation.

IPI		Interim evaluation		New stratification system	
Risk stratification	Score in the new system	Risk stratification	Score in the new system	Combination score in the new system	Risk stratification
LR	0	CR	0	0–1	LR
LIR	1	PR	1	2	IR
HIR	2	SD	3	3	HR
HR	2	PD	3	4–5	VHR

CR, complete remission; HR, high risk; HIR, high-intermediate risk; IPI, International Prognostic Index; IR, intermediate risk; LR, low risk; LIR, low-intermediate risk; PD, progressive disease; PR, partial response; SD, stable disease; VHR, very high risk.

With the use of the combined system, the patients were divided into four independent risk groups: low (LR), intermediate (IR), high (HR), and very high (VHR). The distribution of each group was similar to that for interim evaluation: 152 (48.4%) patients were in the LR, 75 (23.9%) were in the IR, 61 (19.4%) were in the HR, and 26 (8.3%) patients were in the VHR. The 3-year OS rates of the LR, IR, HR, and VHR groups were 96.4%, 86.7%, 46.4%, and 40%, respectively, and the corresponding 3-year PFS rates were 87.1%, 71.5%, 42.5%, and 7.2% (**Figures 1G, H**). Two paired comparisons of PFS were statistically significant (p < 0.05). The OS rates of the HR and VHR groups were marginally significantly different (p = 0.071), and comparisons between any other two groups were significantly different (p < 0.05).

Using this new risk stratification system, we were able to identify the most favorable patients with 3-year OS of >95% and HR and VHR patients with 3-year OS of <50%. Compared with the IPI stratification, the combined system can discern the ~30% of patients with inferior survival (**Table 3**). All of these patients in the new risk stratification system had <50% of 3-year OS. The new risk stratification system can identify ~30% of patients with 3-year OS lower than 50%, compared with ~10% of patients using only interim evaluation data (**Table 3**). Collectively, our results support the use of this new risk stratification system to predict DLBCL patient survival, especially those at high risk of shorter survival.

**Table 3:** Distribution and survival of the patients in the different risk groups.

IPI					Interim evaluation					Combination risk stratification			
Risk group	n (%)	3Y OS%	3Y PFS%		Risk group	n (%)	3Y OS%	3Y PFS%		Risk group	n (%)	3Y OS %	3Y PFS%
LR	135 (43)	93.1	85.1		CR	135 (43)	97.1	84		LR	152 (48.4)	96.4	87.1
LIR	76 (24.2)	86.7	75.1		PR	146 (46.5)	73.9	67		IR	75 (23.9)	86.7	71.5
HIR	68 (21.7)	75.4	51.9		SD	13 (4.1)	47.5	10.3		HR	61 (19.4)	46.4	42.5
HR	35 (11.1)	57.3	46		PD	20 (6.4)	34.1	15		VHR	26 (8.3)	40	7.2

CR, complete remission; HR, high risk; HIR, high-intermediate risk; IPI, International Prognostic Index; IR, intermediate risk; LR, low risk; LIR, low-intermediate risk; OS, overall survival; PD, progressive disease; PFS, progression-free disease; PR, partial response; SD, stable disease; VHR, very high risk.

### Outcome of the Germinal Center B-Cell/Non-Germinal Center B-Cell Subgroups According to Different Risk Stratification Systems

GCB and non-GCB subgroups had been reported with different survival rates according to various studies. We also analyzed the prognosis of GCB and non-GCB subgroups *via* different risk stratification systems.

**Figure 2:**
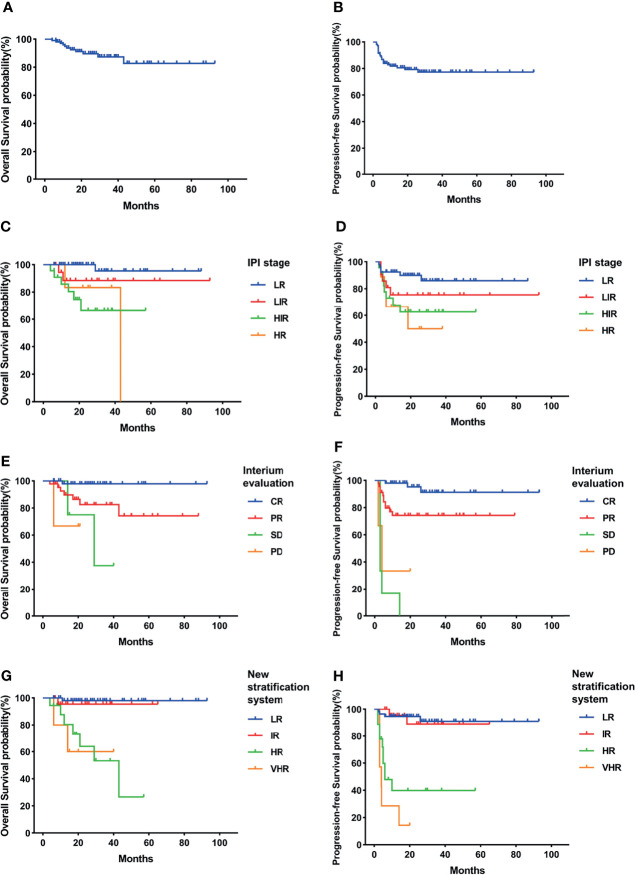
Survival rates of germinal center B-cell (GCB) subgroup. **(A)** Overall survival (OS). **(B)** Progressive-free survival (PFS). **(C)** OS according to International Prognostic Index (IPI) and interim evaluation. **(D)** PFS according to IPI and interim evaluation. **(E)** OS when stratified with interim evaluation. **(F)** PFS when stratified with interim evaluation. **(G)** OS according to the new risk stratification system by combination of interim evaluation and IPI. **(H)** PFS according to the new risk stratification system by combination of interim evaluation and IPI.

**Figure 3:**
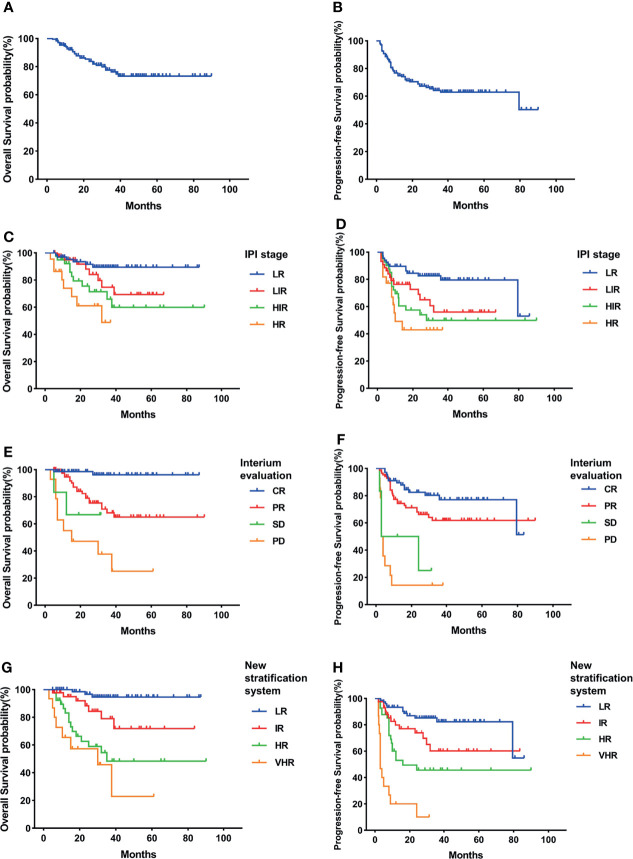
Survival rates of non-germinal center B-cell (non-GCB) subgroup. **(A)** Overall survival (OS). **(B)** Progressive-free survival (PFS). **(C)** OS according to International Prognostic Index (IPI) and interim evaluation. **(D)** PFS according to IPI and interim evaluation. **(E)** OS when stratified with interim evaluation. **(F)** PFS when stratified with interim evaluation. **(G)** OS according to the new risk stratification system by combination of interim evaluation and IPI. **(H)** PFS according to the new risk stratification system by combination of interim evaluation and IPI.

In our study, the GCB group had higher 3-year OS and PFS than the non-GCB group but without statistical difference (OS, 87.4% vs. 76.3%, p = 0.122; PFS, 77.3% vs. 62.8%, p = 0.074) (**Figures 2A, B**, **3A, B**). As in the total cohort, IPI could not separate the HIR and HR groups into the GCB and non-GCB groups (p > 0.05) (**Figures 2C, D**, **3C, D**). And the SD and PD patients in the GCB and non-GCB groups were both with inferior survival than the CR and PR patients (**Figures 2E, F**, **3E, F**), but they were only 9.4% and 11.5%, respectively. In the new risk stratification system, 23.6% of GCB were in the HR and VHR groups and have significantly lower survival than the low- and intermediate-risk groups (**Figures 2G, H**). Compared with the GCB subgroup, the non-GCB subgroup could be precisely discriminated by the new risk stratification system. The OS rates of the HR and VHR groups were not significantly different (p = 0.194), but the OS and PFS comparisons between any other two groups were significantly different (p < 0.05) (**Figures 3G, H**).

## Discussion

DLBCL is a heterogeneous disease with different clinical manifestations, biological features, molecular characteristics, chemotherapy sensitivities, and survival rates. Even though survival was tremendously improved with the advent of immunochemotherapy, there are still 30%–40% of patients who will suffer relapse or refractory disease associated with very poor survival. Researchers have explored how to identify patients at high risk of treatment failure and promptly apply risk-stratified therapy for many years. During the 1990s, the IPI was established by the International Non-Hodgkin’s Lymphoma Prognostic Factors Project based on the clinical characteristics of more than 2,000 patients. All these patients had aggressive NHL and were treated with doxorubicin-containing chemotherapy regimens in western countries (2). The IPI system can identify four risk categories as low, low-intermediate, high-intermediate, and high with 5-year OS rates of 73%, 51%, 43%, and 26%, respectively (2). Because the IPI successfully stratifies four groups with discrete survival curves, it has been widely used for the survival prediction of DLBCL. With the development of immunochemotherapy, the utility of the IPI value for prediction decreased as various studies showed similar survival rates in the HR and HIR groups (17, 18). Since then, new prediction systems based on IPI have been explored.

The NCCN designed the NCCN-IPI system. Compared with IPI, this new stratification system readjusted the influences of age and LDH level. They also limited extranodal involvement to the bone marrow, central nervous system, liver, gastrointestinal tract, and lung. With the use of this system, DLBCL patients can be categorized into four risk groups with 5-year OS rates from 96% in the LR group to 33% in the HR group (9). The NCCN‐IPI approach achieved better discrimination of patient outcomes in the immunochemotherapy era than the original IPI. The NCCN-IPI has also been validated in western and eastern cohorts of *de novo* DLBCL patients, suggesting good reproducibility and accuracy. Huang and colleagues evaluated this system in Chinese patients and demonstrated the superiority of NCCN-IPI over IPI as a prognostic model for DLBCL patients treated with rituximab. However, that study only involved ~100 patients at a single center, which may influence the accuracy of the research (19). In another study by Yang et al., 176 DLBCL patients from one institute did not show significantly different survival between the LR and LIR groups according to the NCCN-IPI stratification (20). Nakaya et al. evaluated the NCCN‐IPI in 284 Japanese DLBCL patients and found that it classified fewer patients as low or high risk (10). Moreover, the NCCN-IPI failed to identify four prognostic groups in the total cohort without upfront autologous hematopoietic stem cell transplantation, as the HR group showed longer 5-year OS and PFS than the HIR group (10). The result was similar to that of another study that found the NCCN‐IPI scores could not categorize DLBCL patients into four risk groups with statistical significance; they were only useful for dividing the cohort into two groups (LR plus LIR and HR plus HIR groups) with significantly different PFS rates (21). Furthermore, the NCCN‐IPI HR group also showed a high PFS rate of 40%–60%, which may need further stratification (11). Collectively, these results indicate that the NCCN-IPI is still not an ideal model to predict DLBCL survival, especially in eastern populations.

Interim evaluation by imaging examination is the standard protocol during DLBCL treatment. Various studies have proved the predictive value of interim evaluation. CT is the most widely used modality. Armitage et al. demonstrated that CR patients with interim CT evaluation had superior survival than others (22). In recent years, ^18^F-FDG PET-CT scans have been increasingly applied for interim evaluation of DLBCL to identify patients with poor prognoses and adopt variable treatment strategies (17). The Deauville 5-point scale was first recommended as the standard reporting tool at the First International Workshop on PET in Lymphoma in 2009. The system compares ^18^F-FDG uptake of the disease lesions with that of the mediastinal and liver pools to calculate scores ranging from 1 to 5 according to the different comparison results. The responses can also be divided into four groups, CR, PR, SD, and PD, by comparing ^18^F-FDG uptake before and after treatment (23). Interim evaluation by PET-CT scans was associated with prognosis by multiple retrospective (24–27) and prospective (28, 29) studies. However, the results also showed that the positive interim PET-CT scans were not strictly correlated with prognosis, as a substantial number of patients could still experience prolonged survival in remission (28–30). Kurch and colleagues also reported that the positive predictive value was significantly lower for the Deauville score than the ratio of the uptake values (Δstandardized uptake value max) for before treatment and interim evaluations (31). Another group also published results showing that the interim Deauville 5-point scale was not associated with the survival of patients with DLBCL (32). These observations call into question the application of interim evaluation results as a single parameter for DLBCL risk stratification.

In our study, both IPI and interim evaluation could stratify the patients into three groups with different OS and PFS rates. However, the IPI cannot identify a truly high-risk group, as all groups’ OS curves were >50%. When we stratified with interim evaluation, we could discern a subset of HR patients with 3-year OS of <30%, but the percentage was only ~10%. This approach failed to identify some HR patients. To improve stratification efficacy, we integrated the IPI and interim evaluation data. The new system identified a high percentage of LR patients with a favorable prognosis of 3-year OS of >95% and also discerned ~30% of patients with 3-year OS of <50%. The result may indicate that LR patients can be successfully treated with traditional immunochemotherapy, while the HR and VHR patients identified with this new risk stratification system should be prioritized for clinical trials due to their poor survival.

## Conclusions

The present study confirmed the improved discriminatory capacity of the new stratification system in patients with DLBCL. Both parameters in the new system are based on daily clinical practice without additional expensive examinations. It is easily applied and accepted by patients and physicians. The new stratification system may help identify the best potential populations for clinical trials, although this needs further exploration.

## Data Availability Statement

The raw data supporting the conclusions of this article will be made available by the authors, without undue reservation.

## Ethics Statement

The studies involving human participants were reviewed and approved by Shandong Provincial Hospital affiliated to the First Medical University of Shandong, the Affiliated Hospital of Qingdao University, the Affiliated Yantai Yuhuangding Hospital of Qingdao University, and Dongying People’s Hospital. The patients/participants provided their written informed consent to participate in this study. Written informed consent was obtained from the individual(s) for the publication of any potentially identifiable images or data included in this article.

## Author Contributions

XShi, XQL, XML, YHL, HC, and XF contributed to the study design, analysis, and writing of the manuscript. YL, SH, YZ, XZ, and XW contributed to the data analysis and interpretation. DL and XSun contributed to the data collection and preparation of the manuscript. All authors contributed to the article and approved the submitted version.

## Funding

This study was supported by the National Natural Science Foundation (No. 81270598, No. 81473486, and No. 81770210), Key Research and Development Program of Shandong Province (No. 2018CXGC1213), Technology Development Projects of Shandong Province (No. 2017GSF18189, No. 2016GSF201029), Technology Projects of Jinan (No. 201704092, No. 202019044), Taishan Scholar Foundation of Shandong Province, Shandong Provincial Engineering Research Center of Lymphoma, and Key Laboratory for Kidney Regeneration of Shandong Province.

## Conflict of Interest

The authors declare that the research was conducted in the absence of any commercial or financial relationships that could be construed as a potential conflict of interest.

## Publisher’s Note

All claims expressed in this article are solely those of the authors and do not necessarily represent those of their affiliated organizations, or those of the publisher, the editors and the reviewers. Any product that may be evaluated in this article, or claim that may be made by its manufacturer, is not guaranteed or endorsed by the publisher.
